# 

**Published:** 1892-01

**Authors:** 


### TABLE OF CONTENTS.

ORIGINAL COMMUNICATIONS. PAGE

Articulation of the Teeth. By ISAAC B. DAVENPORT, M.D., M.D.S., Paris, France . . . . . 1

Obscure Pains in and about the Jaws. By J. E. GARRETSON, M.D., D.D.S., Philadelphia . . . . . 8

A Study in the Regulation of Teeth. By RODRIGUES OTTOLENGUI, M.D.S., New York . . . . . 18

Some Thoughts upon Personal Dental Education. By W. MITCHELL, D.D.S., London, England . . . . . 26

Dental Medicine. By R. M. CHASE, D.D.S., M.D . . . . . 32

Ethical Environments of the Dental Surgeon. By ALBERT WESTLAKE, D.D.S. . . . . . 36

REPORTS OF SOCIETY MEETINGS.

New York Odontological Society . . . . . 41

American Dental Association . . . . . 50

Odontological Society of Pennsylvania . . . . . 60

EDITORIAL.

Retrospective and Prospective . . . . . 70

Dentists and the American Medical Association . . . . . 71

A Legal Outrage . . . . . 74

BIBLIOGRAPHY . . . . . 75

OBITUARY.

Dr. Charles A. Kingsbury, Philadelphia Dental Club . . . . . 76

J. Francis Foltz, D.D.S. . . . . . 77

CURRENT NEWS.

Anniversary Meeting of the First District Dental Society of New York . . 77

First District Dental Society, New York—Special Announcement . . . . 79

American Academy of Dental Science . . . . . 79

Items . . . . . 80

